# Establishing cross-systems collaborations for implementation: protocol for a longitudinal mixed methods study

**DOI:** 10.1186/s13012-020-01016-9

**Published:** 2020-07-16

**Authors:** Alicia C. Bunger, Emmeline Chuang, Amanda Girth, Kathryn E. Lancaster, Fawn Gadel, Marla Himmeger, Lisa Saldana, Byron J. Powell, Gregory A. Aarons

**Affiliations:** 1 College of Social Work, The Ohio State University, 1947 College Road, Columbus, OH 43210 USA; 2 School of Social Welfare, University of California Berkeley, 120 Haviland Hall, Berkeley, CA 94720 USA; 3 John Glenn College of Public Affairs, The Ohio State University, 1810 College Road, Columbus, OH 43210 USA; 4 College of Public Health, The Ohio State University, 1841 Neil Avenue, Columbus, OH 43210 USA; 5 Public Children Services Association of Ohio (PCSAO), 37 West Broad Street, Suite 1100, Columbus, OH 43215 USA; 6 Oregon Social Learning Center, 10 Shelton McMurphey Blvd., Eugene, OR 97405 USA; 7 Brown School, Washington University in St. Louis, One Brookings Drive, St. Louis, MO 63130 USA; 8 UC San Diego, Department of Psychiatry, 9500 Gilman Drive (0812), La Jolla, San Diego, CA 92093 USA; 9 UC San Diego Dissemination and Implementation Science Center, La Jolla, CA 92037 USA

**Keywords:** Cross-system interventions, Collaboration, Implementation strategies, Child welfare, Substance use treatment

## Abstract

**Background:**

Cross-system interventions can help integrate services across different service delivery systems but require organizations to establish strong collaborative relationships for implementation. Contingency theory suggests that the effectiveness of different collaborative strategies (i.e. specific ways organizations align operations and services) varies by context. This paper describes a study of different strategies for fostering collaboration between child welfare and substance abuse treatment agencies and the conditions under which they are effective for implementation. We also describe the development and piloting of the Collaborating Across Systems for Program Implementation (CASPI) tool—a decision-making guide intended to help researchers and organizational leaders identify and use appropriate collaborative strategies for their context.

**Methods/design:**

This multisite longitudinal, mixed methods study, leverages a naturally occurring implementation initiative -- in up to 17 Ohio counties -- to implement Ohio START (Sobriety Treatment and Reducing Trauma). START is a child welfare model that requires strong collaboration with local substance use treatment organizations to promote integrated services. During the first two years, we will identify collaborative strategies associated with improved START implementation (penetration and fidelity) and service delivery outcomes (timeliness), given system, and organizational features. We will conduct a convergent mixed methods study drawing on worker surveys, agency documents, administrative data, formal partner agreements, and group interviews. Data will be integrated and analyzed using Qualitative Comparative Analysis (QCA). To develop the CASPI, an expert panel comprised of implementation experts, and community stakeholders will convene to synthesize our findings and develop contents (including a decision tree). During the final year of the study, we will assess the acceptability, appropriateness, and feasibility of the CASPI in a randomized vignette experiment, and a pilot-test with 3 child welfare agencies that have not yet implemented START.

**Discussion:**

Our results will lay the groundwork for a larger controlled trial that will test the CASPI’s effectiveness for supporting effective and efficient implementation of cross-system interventions like START. The CASPI is expected to help leaders and researchers select and use collaboration strategies tailored to their context and be applicable in a wide range of settings including rural communities. Our work also advances system-level implementation strategies.

**Trial registration:**

NCT03931005, Registered April 29, 2019.

Contributions to the literature

- Implementing cross-system interventions depends on strong collaboration. Yet, collaborative strategies vary and the conditions under which they are most effective for implementation are unclear.
- This study will (1) examine the collaborative strategies associated with implementation of a cross-system intervention that aligns child welfare and substance use treatment systems, (2) develop the Collaborating Across Systems for Program Implementation (CASPI) tool, a flexible decision support for organizational leaders, and (3) pilot test CASPI with organizational leaders.
- Our study is intended to advance the effectiveness and efficiency of implementing cross-system interventions by helping leaders identify challenges, and effective -collaborative strategies that address them.

## Background

Families with complex health and human service needs rely on services delivered by multiple systems, but they experience fragmented or poorly coordinated care [1]. Cross-system interventions align services across systems - screening and assessment in one system, followed by referral and treatment in another can reduce fragmentation and coordinate care [2, 3]. However, cross-system interventions can be difficult to implement because organizations from different systems must work together for effective implementation and service delivery [4]. We define *collaboration strategies* as the specific ways organizations align services and operations and consider them necessary strategies for implementing cross-system interventions. Collaboration strategies can vary substantially; partners might co-locate services, jointly fund specialized staff, create streamlined referral processes, contract for expedited service access or a specific type of treatment, share case information/data, or establish cross-agency teams [5–8]. Organizations may choose to work with one or many partners and might codify these strategies in a formal agreement (e.g. a memorandum of understanding or contract) or execute them informally. While successful implementation of cross-system interventions depends on strong collaboration, the field lacks a clear understanding of variations in collaboration strategies, robust evidence of their effectiveness under different conditions, and tools that allow researchers and agency leaders to use these strategies effectively [9].

This study will advance implementation of cross-system interventions by examining collaborative strategies, and the conditions under which they are effective. We leverage a naturally occurring implementation initiative in 17 Ohio counties to implement Sobriety Treatment and Recovery Teams (START), a national child welfare model that requires strong cross-system collaboration with local substance use treatment organizations to promote expedited and integrated services [10, 11]. The central hypothesis is that child welfare agencies develop and expand partnerships with substance use treatment organizations to align their operations and front-line workforce practices across systems. This alignment has potential to support START implementation and improve service access and outcomes for families. However, agencies might select different types of collaborative strategies depending on the system and organizational features. We will draw on these results and develop the Collaborating Across Systems for Program Implementation (CASPI) tool, a decision-making guide for agency leaders responsible for initiating collaboration. The specific aims are to:

- Aim 1: Examine cross-system collaborative strategies associated with START implementation (penetration and fidelity), and service delivery outcomes (timely treatment), given contextual features.
- Aim 2: Specify cross-system collaborative strategies for implementation and develop a flexible collaboration decision-support guide (CASPI).
- Aim 3: Assess the acceptability, appropriateness, and feasibility of the collaboration decision-support guide (CASPI).

### Study context – Ohio START

As a result of the national opioid crisis, child welfare systems have reported an increase in the number of children in foster care as parental substance use puts children at greater risk of maltreatment [12]. Ohio has been hard hit by the opioid epidemic with the highest rates of heroin and synthetic opioid-related deaths in the country [13, 14]. In recent years, 50% of all the children removed to state custody in Ohio were due to parental substance use, and about half of those children (28% of all children brought into custody) had parents who used opioids [15]. An associated 11% increase in the overall number of children in custody [15] placed substantial strain on Ohio’s 85 public child welfare systems^1^ especially in the state’s rural southern counties. Child welfare systems are well positioned to help parents with substance use disorders. Promising evidence-based cross-system interventions exist that emphasize brief screening and linkage to effective treatment in the addiction services system, including the national START model (Sobriety Treatment and Recovery Teams) [10, 11], which has been registered as a best practice in the California Evidence Based Clearinghouse for Child Welfare [16].

START creates a coordinated pathway to substance use treatment for parents through a series of stages. Parents first are screened for substance use disorders, then linked with a family peer mentor (a peer recovery supporter with lived child welfare and recovery experience) who engage and support parents. Next, parents are referred by the child welfare worker to a substance use treatment provider in the community for assessment, and at least four treatment sessions (intensive outpatient or other type of needed treatment as indicated by the assessment) within 28 days of entering the child welfare system. Meanwhile, child welfare workers, family peer mentors, substance use treatment providers, and the parents team up to align case goals, and service timelines to support parents as they work toward sobriety and reunification within mandated child welfare timelines [17–19]. START has been shown to expedite parent’s access to and completion of treatment and increase their use of medication for opioid use disorders (MOUD). Subsequently, parents who received START improved their likelihood of reaching sobriety and reunifying with their children, and reduced subsequent maltreatment risk [10, 11, 20, 21].

**Fig. 1:**
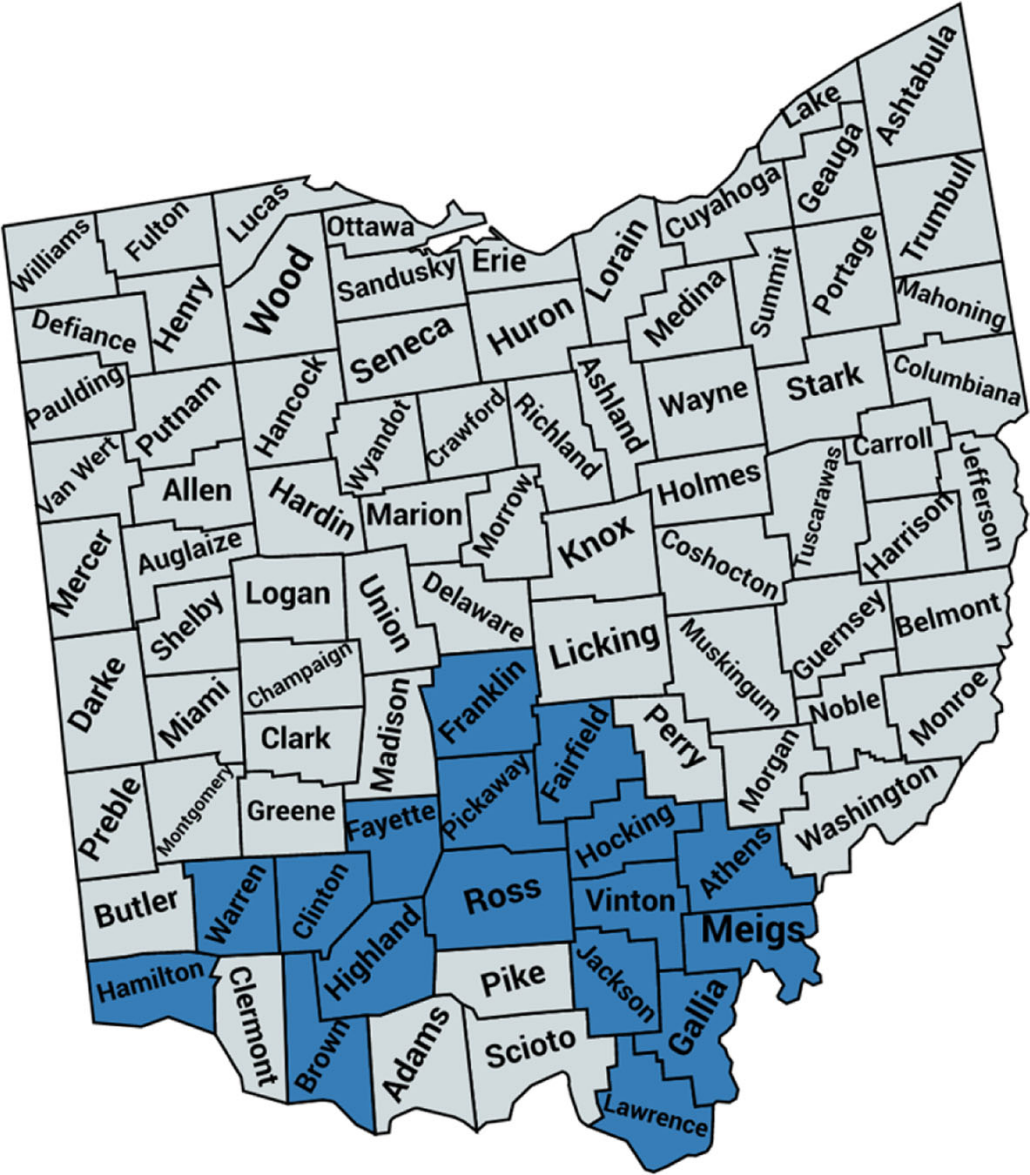
Map of Ohio START, Counties (Cohort 1; n=17)

START was adopted in Ohio in early 2017, locally adapted to respond to trauma exposure (a trauma screening component was added) and renamed as the Ohio Sobriety Treatment and Reducing Trauma program. Ohio START is led by and housed at the Public Children Services Association of Ohio (PCSAO), a statewide non-profit organization that represents 85 county-based public child welfare agencies. With grant funding from the state of Ohio and several foundations, PCSAO designed and organized a standardized approach to training and implementation supports which are targeted to county-systems given the decentralized structure of child welfare services in the state. Ohio START implementation began in March 2017 in 17 county systems located in the southern region of the state where opioid overdose death rates were highest [14], and began serving families through the program in early 2018 (Fig. 1). These counties include a mix of rural (n=9) and urban (n=8) regions; ten counties are also considered Appalachian thus reflecting the diversity of regional contexts for implementing new system-level interventions. (See Supplemental File 1 for details about Ohio START design and implementation).

### Collaborative strategies for implementing START and other cross-system interventions

Implementing START depends on strong collaboration between child welfare agencies and at least one local substance use treatment provider [22]. Child welfare agencies often depend on substance use treatment organizations to hire and co-supervise the family peer mentor, provide expedited access to treatment, align case plans, participate in family team meetings, and share data on parents’ progress in treatment. However in earlier tests of START, collaboration also was the biggest implementation barrier [23]. Formal administrative-level partnerships negotiated among organizational leaders were necessary to provide support for effective collaboration, and implementation with fidelity at the front-lines [21, 24]. Working through these challenges and setting up formal agreements took time, delaying implementation [23]. The resulting collaborations among child welfare and substance use treatment partners varied across counties [23, 25, 26] suggesting that the most effective approaches to collaboration for implementing START might depend on context. Although successful implementation of START (and other types of cross-system interventions) is contingent on strong cross-systems collaboration [22], the field lacks clear descriptions of cross-system collaborative strategies, robust evidence of their effectiveness under different conditions, and tools that allow researches and agency leaders to use these strategies effectively.

### Conceptual model

**Fig. 2:**
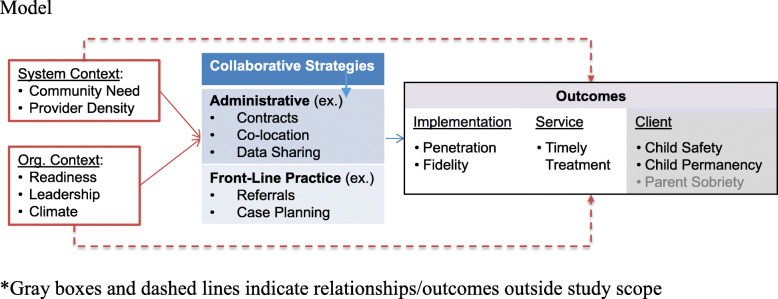
Conceptual Model, *Gray boxes and dashed lines indicate relationships/outcomes outside study scope

Our study is anchored by a conceptual model informed by frameworks related to contextual determinants of implementation, and contingency theories of organizational design that explain strategy selection and effectiveness (Fig. 2). *Collaborative strategies refer to the specific methods used to align operations and services and are used at two-levels within a system.* We argue that collaborative strategies are essential implementation strategies for cross-system interventions. At the *administrative-level*, child welfare leaders must establish a formal partnership with a substance use treatment provider by identifying a partner provider and negotiating how they will align their organizations (e.g. by co-locating, sharing data) [8, 27]. Administrative-level partnerships support collaboration at the *front-lines* where practitioners engage in a different set of collaborative strategies (e.g. referring parents, aligning case plans) [25]. Strong collaboration with public child welfare agencies has been associated with positive effects on private organizations’ programming and performance [28]. However, the collaborative strategies that work well in one county, might not work in another. For instance, a child welfare agency might choose a substance use treatment partner that employs a family peer mentor (a component of the Ohio START model), and contract with them for services. However, another agency located in an area with fewer substance use treatment providers who do not yet employ family peer mentors might need to partner with a provider to jointly develop, fund, recruit for, and supervise this position. There is likely no one “best” way to collaborate for implementation, although both agencies in this example would need to engage in negotiation and resolution in order to address contextual barriers [29].

To understand contextual determinants, we draw on the Exploration, Preparation, Implementation, and Sustainment (EPIS) framework, where external system and internal organizational contextual determinants feature prominently throughout implementation phases in public systems [30]. EPIS “bridging factors” including collaboration, contracting, and formalized agreements can link outer and inner context as well as connect interorganizational networks operating in state or county systems [31]. To understand collaborative strategies, we draw on *contingency theories of organizational design* which explain how the effectiveness of the collaborative strategy depends on alignment with the environment [32, 33]. In particular, collaborative governance frameworks explain how the *system context* (e.g. community needs and availability of treatment partners) creates opportunities and incentives specifically for different collaborative strategies [34, 35]. We also know from the *Theory of Implementation Effectiveness* [36, 37] that the selection and effectiveness of organizational strategies is determined by *organizational context* (e.g. leadership, readiness, and climate), which may influence how effectively executive leaders support front-line collaboration (e.g. referrals, case planning) .

We draw from these determinant and contingency theories to hypothesize that the interaction of the system context and organizational context determine the specific *collaborative strategies* that emerge, and their effectiveness. We expect that strategies that optimize fit will lead to better implementation outcomes, especially high rates of *penetration* and *fidelity* to the elements of the START model that require collaboration (e.g. working with the family peer mentor, referrals, treatment receipt, and family team meeting participation) [38]. Effective implementation will generate better *service outcomes* (e.g., timeliness), and ultimately *child and family outcomes* (safety, permanence, sobriety; although a full test of outcomes is beyond the scope of this study).

## Methods/design

### Aim 1: examine collaborative strategies associated with START implementation and service delivery outcomes given context

#### Design

The objective of this aim is to identify the types of collaborative strategies associated with implementation and service outcomes, and the contexts under which they are effective. Aim 1 uses a convergent mixed methods design [39] that draws on qualitative data from each county (formal agreements and small-group interviews) to capture collaborative strategies and multiple quantitative data sources (Table 1) to measure context, and outcomes (implementation and service) consistent with our conceptual model (Fig. 2). We will integrate data streams during analysis using a variant of qualitative comparative analysis (known as fuzzy-set qualitative comparative analysis, fsQCA) [44, 45].

**Table 1:** Aim 1 Quantitative constructs and measures by data source (measured at county-level)

Construct	Measures	Timing
**Existing Public Health Data**		
Community Need	• County rates of child abuse & neglect	T1
	• County rates of opioid-related overdose death	
	• County rates of naloxone administration	
**SAMHSA Behavioral Health Treatment Locator**		
Provider Density	• # of organizations that deliver substance use tx in county	T1
	• # of different MOUD available in county	
**Worker Surveys**		
Readiness	Organizational Readiness for Implementing Change (ORIC) [40], 10 items Subscales: 1) Commitment (5 items); 2) Efficacy (5 items) 5 point scales (1=disagree, 5=agree)	T1
Leadership	Implementation Leadership Scale [41]; 12 items total Subscales: 1) Proactive (3 items); 2) Knowledgeable (3 items); 3) Supportive (3 items); 4) Perseverant (3 items) 5 point scales(0=not at all; 4=very great extent)	T2-T3
Climate	Climate Measure [42]; 6 items total Subscales: 1) Expected (2 items); 2) Supported (2 items); 3) Rewarded (2 items) 5 point scales (1=disagree, 5=agree)	T2-T3
Front Line Collaboration	• Referral frequency to substance use treatment partner 6 point scale (1=Not once, 5=Daily)	T2-T3
	• Wilder Collaboration Factors inventory [43]; 40 items total Subscales: 1) Environment (6 items); 2) Membership (6 items); 3) Process/Structure (13 items); 4) Communication (5 items); 5) Purpose (7 items); 6) Resources (3 items) 5 point scales (1=strong disagree, 5=strongly agree)	
**OSU Needs Portal**		
Penetration	• Number of cases served	Ongoing
	• % of cases with a family peer mentor visit	
	• % of cases with at least one substance use treatment logged	
Fidelity	% of parents who: • Receive substance use screening	Ongoing
	• Receive at least one treatment session (if screened in)	
	• Participate in at least one family team meeting (if screened in)	
Timeliness	Average number of days between entering the child welfare system and:	Ongoing
	• Substance use disorder screening	
	• First Family peer mentor visit	
	• First treatment session	

### Qualitative data

#### Formal agreements

Formal collaborative relationships often are codified via contracts, memorandums of understanding (MOU), or other types of interagency agreements. We expect most child welfare agencies have at least one formal agreement with a substance use treatment provider for START. The study PI (Bunger) will gather electronic copies of formal agreements executed with behavioral health service providers for Ohio START via email from each child welfare agency director or main program contact. These high-level administrators often are directly involved in negotiating and monitoring partnerships, are familiar with, and have access to these agreements. Members of the research team led by an expert in contracting (Girth) will conduct a content analysis. Specific details about each partnership will be extracted and coded including: type of agreement, provider roles and responsibilities, performance expectations, data sharing expectations, and specificity of partnership terms. Formal agreements will be coded both iteratively--generating themes as they emerge--and structured using elements of the START Implementation Manual pertaining to partner agreements [46]. Two coders will review each formal agreement to increase reliability.

#### Interviews

To capture the range of collaborative actions, we will collect qualitative information about collaboration between substance use and child welfare partners via two semi-structured interviews in each county. We will conduct at least one interview in each county with child welfare stakeholders and at least one interview with a substance abuse treatment provider identified by the child welfare agency as a key partner in START implementation (minimum 2 interviews for each of 17 counties, for a total of 34 interviews). Interviews will be conducted using a small-group format (two or three key informants in each interview). Small-group interviews are more efficient than individual interviews for gathering multiple perspectives, while providing a deeper interpretive lens than a focus group [47]. These interviews will inform collaborative strategy specification [9] (e.g. target, temporality, justification) and identify other salient contextual features.

##### Participants

We estimate interviewing at least 68 individuals (two participants in each of the 34 interviews) who represent agency leaders, supervisors, front-line workers, family peer mentors, or other stakeholders from the child welfare and substance use treatment agencies who are directly involved in START implementation. We will work with our partners at PCSAO to identify and invite participants. Participants will be offered a $30 gift card as an incentive for their participation.

##### Data collection

Sixty-minute group interviews will be conducted by phone (to accommodate professionals’ busy schedules) by at least two members of the research team (a facilitator and co-facilitator). All research team members are masters’ or doctoral level students/researchers who have been trained and supported by senior members of the team with qualitative interviewing expertise. We will follow a semi-structured interview guide asking respondents to reflect on the current collaborative partnership between the child welfare and substance use treatment organizations to implement START, specifically: (1) how the partnership formed, (2) the strategies used to collaborate (how) and the rationale for the strategies chosen, (3) strengths and challenges of collaboration, (4) the role of external coordinating entities in the region, and (5) participant demographics. Understanding how and why key stakeholders perceived particular collaborative strategies as working can clarify potential mechanisms [48, 49]. Interviews will be audio recorded and professionally transcribed; interview facilitators and co-facilitators will prepare written reflections about issues that emerged in each interview about collaboration and implementation.

##### Coding/analysis

Interviews are intended to generate rich descriptions of collaborative strategies, and explanations for their use – transcripts will be analyzed using a template analysis approach, a type of thematic analysis to rigorously and succinctly summarizing and reducing data [50]. We will draw on the concepts in our conceptual model (types of collaborative strategies, rationale, and contextual determinants), and themes that emerge in our reflections to develop an initial codebook. We will refine the codebook in iterative cycles where two coders will independently apply it to a subset of transcripts, compare codes, discuss discrepancies (with a third coder), and refine codes. Two independent coders will apply final codebook, and meet to resolve discrepancies on transcripts with less than 80% agreement. Data will be summarized into a county (rows) matrix that combines thick description of the types of collaborative strategies used and their rationale, with summary information about the contextual determinants (columns) to support cross-case comparison and identification of patterns.

### Quantitative data sources

#### Existing public health data on county needs

County rates of child maltreatment, will indicate community needs for child welfare intervention and will be extracted from the PCSAO 2019 Factbook [15]. The Factbook is updated bi-annually and draws on administrative data from all county child welfare agencies compiled by the Ohio Department of Job and Family Services (ODJFS) and verified with each county agency director for accuracy. County rates and case numbers of opioid-related overdose deaths will serve as a proxy for community need for substance use treatment due to opioid use disorders. Opioid-related overdose death rates will be drawn from routine surveillance data from the Ohio Violent Death Reporting System of the Ohio Department of Health (ODH) [14]. Opioid-related overdose death estimates will also be augmented with naloxone administration data obtained from the Ohio Department of Public Safety, Division of Emergency Medical Services.

#### SAMHSA behavioral health treatment locator

Information on substance use treatment availability in each county will be gathered the SAMHSA Behavioral Health Treatment Locator (findtreatment.samhsa.gov). This national directory is based on data gathered via the National Survey of Substance Abuse Treatment Services (NSSATS), with all facilities reported by state behavioral health authorities. A member of the research team will conduct a county-level search and download records for identified substance use treatment providers and buprenorphine practitioners. Because the SAMHSA treatment locator might be incomplete [51], we will consult local resource directories to identify additional providers. We will generate two indicators of treatment availability: (1) the number of providers that deliver substance use treatment in each county (as a proxy of treatment availability), and (2) the number of different types of MOUDs (e.g., Methadone, Buprenorphine) in the county, where higher numbers indicate a fuller spectrum of MOUD availability.

#### Worker surveys

As part of a local evaluation of Ohio START, surveys were administered to child welfare staff to assess the context for implementation after training (Time 1, October 2017), ten months later during implementation (Time 2, August 2018), and about one year later (Time 3, October 2019). These data will be leveraged for this study, with child welfare agencies’ permission.

##### Participants and data collection

Participants include approximately 150 child welfare workers and project-associated personnel: intake workers, ongoing caseworkers, referral specialists, family peer mentors, unit supervisors, and agency leaders. This estimate accounts for current personnel (*n* = 100), and those who began working for the child welfare agencies between the time of the baseline and follow-up surveys, given high workforce turnover [52]. Each child welfare agency provided a roster of START-involved staff and their email addresses before each survey administration. Surveys were administered online to all identified individuals.

##### Key constructs and measures

The surveys assessed four organizational constructs; individual responses within each child welfare agency will be aggregated (Table 1).

- Readiness for Implementation: Participants report on their perceptions about the child welfare agency’s readiness at all three time points via the Organizational Readiness for Implementing Change (ORIC) scale [40].
- Implementation Leadership: The Implementation Leadership Scale (ILS) measures the degree to which those in leadership positions support or hinder START implementation [41] and has been validated in child welfare, substance use disorder treatment, and mental health treatment settings [53, 54]. The ILS is administered at Times 2 and 3 since the items ask workers to reflect on their leaders’ behaviors during implementation.
- Implementation Climate: The implementation climate, or degree to which START is expected, supported, and rewarded is assessed using the implementation climate scale [42]. The climate scale items ask workers to reflect on their experience using START and will be measured at Times 2 and 3.
- Front-Line Collaboration: The degree to which front-line practitioners are engaging across child welfare and substance use treatment organizations will be assessed in two ways. One survey item measures *referrals* (at all three time points): where workers list the organizations to which they refer parents for substance use treatment within the past 6 months, and the frequency of their referrals. Second, the quality of front-line collaboration is measured at Time 3 using the Wilder Collaboration Factors Inventory [43, 47].

#### County Fidelity/service tracking - OSU needs portal

Child welfare agencies involved in Ohio START are required to track and report on substance use screenings, family peer mentor contacts, treatment dates, and dates of family team meetings. The OSU Needs Portal is a web-based system designed to manage child welfare workers’ referrals [55, 56] that has been adapted for collecting START fidelity and service data, and rolled out in all 17 child welfare agencies. Aggregated data are available publicly (https://u.osu.edu/ohiostart/evaluation/dashboard/).

##### Key constructs and measures

County-level data that reflects two implementation (penetration and fidelity) and one service outcome (timeliness) will be extracted from the needs portal.

- Penetration: Penetration reflects the reach, or the degree to which START is used within each county [38]. Indicators will include the: (1) number of cases (families) served, (2) percentage of cases with a family peer mentor visit, and (3) percentage of cases with at least one substance use treatment visit logged in the Needs Portal.
- Fidelity: Fidelity reflects the degree to which START’s collaborative components are implemented as intended [38] and our study focuses on adherence. Three indicators will be extracted for each county: the percentage of (1) parents who received a substance use screening, (2) screened-in parents who received at least one treatment session, and (3) screened-in parents who participated in at least one family team meeting with a family peer mentor.
- Timeliness: START should expedite parents’ access to treatment after initial screening. Timeliness will be measured as the average number of days between entering the system and (1) substance use screening, (2) family peer mentor visit, and (3) the first treatment session.

### Analysis – integrating quantitative and qualitative data using QCA

Quantitative and qualitative data will be integrated to examine combinations of *conditions* (community need, leadership, readiness, implementation climate, collaborative strategies) associated with better implementation (penetration, fidelity) and service outcomes (timeliness) consistent with our conceptual model (Fig. 2). With the county system (n=17) as the unit of analysis, we will use a configurational comparative technique known as fuzzy-set Qualitative Comparative Analysis (fsQCA) [45, 57]. QCA is well suited for our study because instead of treating each condition as an independent predictor, it allows for identification of different combinations of conditions associated with an outcome. This approach is useful for identifying combinations of implementation strategies associated with innovation uptake [58].

First, data on each condition and outcome will be calibrated as an interval level measure between 0 and 1 [44, 45]. A truth table will be constructed that delineates all possible combinations of conditions (types of collaboration strategies) and outcomes (penetration, fidelity, services) [57]. These combinations will be compared to determine *consistency,* or the extent to which counties with similar combinations of conditions experience the same outcomes, and *coverage,* or the extent to which an outcome is explained by similar combinations of conditions. A key focus will be on identifying combinations of conditions and particularly collaborative strategies that might prove necessary vs. sufficient for high START implementation. All analyses will be conducted using the QCA package in R. In the event that we cannot reach minimum consistency or coverage levels in the QCA model, or experience limited variation in outcomes across the 17 counties (e.g., none of the counties implement START successfully), we will engage in cross-case comparison (e.g., qualitative examination of similarities and differences between higher or lower implementing counties) and/or thematic analysis of qualitative interviews to identify collaborative strategies that best facilitate START implementation.

### Aim 2 - specify cross-system collaborative strategies and develop the CASPI, a decision-support guide.

The *objective of this aim* is to specify collaborative strategies identified in Aim 1, and develop the CASPI, a decision-support guide that will inform collaborative strategy selection to support scale up of cross-system interventions like START. We will use a multidisciplinary and participatory process to develop organizational and system-level implementation strategies [59, 60]. The CASPI developed in this aim will inform collaborative strategy selection for researchers and practitioners implementing cross-system interventions.

### Preparation

First, we will create a table of the administrative collaborative strategies linked to each contextual condition drawing on (1) the configural patterns of conditions, strategies, and outcomes from Aim 1, and (2) themes that emerge from the group interviews. This table, and a summary will be distributed to an expert panel.

### Expert panel

Next, a multi-disciplinary expert panel comprised of the research team, and partners from the child welfare and behavioral health community will convene for a two-day working meeting in Year 2 to accomplish two goals. First, the panel will specify the collaborative strategies drawing on evidence from Aim 1 (and professional experiences) using Proctor and colleagues’ guidelines [9]. We will describe the contextual conditions under which each strategy is effective as well drawing from our Aim 1 results.

Second, the panel will make recommendations for CASPI contents and packaging. One of the primary contents will be a decision analysis tool/tree to walk leaders through a series of smaller, more manageable if/then scenarios to guide collaborative-strategy decision making under different contextual conditions that emerge from Aim 1 results [61]. Consistent with our general contingency theory approach, we anticipate that the decision analysis tool might prompt directors to consider contextual conditions (e.g. the number of potential partners and their services, whether there is an existing partnership), that lead them to recommended collaborative strategies. The panel will recommend and develop additional CASPI contents (Table 2). After the meeting, the panel will revise, review, and refine the contents.

**Table 2:** Collaborating Across Systems for Program Implementation (CASPI) Contents

Anticipated Contents	
• 2 page brief that describes Aim 1 results in lay language (geared for busy professionals),	
• Specified descriptions of collaborative strategies	
• Decision analysis tool that guides selection of collaborative strategies given context	
• Sample language that specifies the nature and expectations that could be included in contracts, MOUs, data use agreements, etc.	

### Aim 3 - assess the acceptability, appropriateness, and feasibility of the CASPI.

Using a sequential mixed-methods approach where quantitative methods occur first followed by qualitative methods that receive more emphasis (i.e., quan-QUAL) [39], we will assess the CASPI’s acceptability, appropriateness, and feasibility. This will coincide with statewide Ohio START expansion. In the first quantitative phase, a randomized vignette experiment will examine the CASPI’s acceptability, appropriateness, and feasibility. Second, we will pilot the CASPI and conduct descriptive case studies with three county child welfare agencies to refine the CASPI. Data will be integrated during interpretation. The results of this aim are expected to provide preliminary feasibility evidence of the CASPI, in preparation for a subsequent trial.

### Phase 1 - vignette experiment (quantitative)

This first phase involves a randomized vignette experiment using a classical two-group parallel design comparing the acceptability, appropriateness, and feasibility of CASPI with general collaboration supports (e.g. a list of collaborative implementation strategies and their definitions) (see Supplemental File 3 for CONSORT checklist). Because the CASPI will offer a structured approach to tailoring and applying collaborative strategies based on the context, we expect agency leaders (the anticipated users) to rate the CASPI as more acceptable, appropriate, and feasible than general collaboration support.

#### Participants, assignment, and conditions

With help from PSCAO, we will recruit via email an executive leader (e.g. individual employed as executive directors, or primary programmatic contact) from all 85 Ohio child welfare agencies to participate in this phase. Given the high success rate of engaging agency directors in other PCSAO surveys, we anticipate an 80% response rate (about 68 participants). Leaders will be recruited from agencies that are both implementing (about 50% of agencies by the anticipated start date of this phase given the rolling cohort approach to START implementation in Ohio) and those that have not yet implemented START to ensure a diversity of implementation experiences are represented in the sample.

Participants who volunteer will be sent a link to an online survey that asks them to respond to a vignette about a child welfare agency leader tasked with selecting, negotiating, and executing a partnership with a behavioral health organization. Participants will be randomly assigned using simple randomization procedures to one of two groups (using the randomizer element in Qualtrics, which conceals the allocation sequence to the research team). In the experimental group vignette, participants will be directed to the full CASPI (provided in its entirety online) as a potential tool that the hypothetical vignette leader could use to support their decision making. In the control group vignette, participants will be directed to information about general collaboration supports (e.g., a list of collaborative implementation strategies and their definitions). Participants and research team members will be blinded to the study condition at the time of assignment.

#### Measures and analysis

After reading through descriptions of START and collaboration supports (either the CASPI or general supports), participants will be asked 12 questions (in an online survey) intended to measure three primary outcomes: the acceptability (perceived satisfaction), appropriateness (perceived fit or compatibility), and feasibility (perceived utility) of the CASPI or general collaboration supports [38] using validated scales (Acceptability of Intervention Measure (AIM), Intervention Appropriateness Measure (IAM), and the Feasibility of Intervention Measure (FIM), respectively) [62]. Each scale contains four items, with 5-point Likert rating scales. We also will ask participants to report on basic demographics (gender, race, ethnicity, experience in the field) and the names of up to five substance abuse organizations with which their organization has a formal MOU or contract. These responses will be used to identify PCSAs for recruitment in the qualitative phase.

We will compare the experimental and control group participants’ AIM, IAM, and FIM scores using independent-samples t-tests (or nonparametric alternatives) to assess the CASPI’s acceptability, appropriateness, and feasibility relative to general collaboration support. We will also use regression to explore whether participants’ ratings of CASPI and general support tools vary across demographic group, or with the number of existing formal partnerships. These secondary analyses will identify whether there are conditions for which the CASPI should be refined.

### Phase 2 - pilot test and descriptive Case studies (qualitative)

#### Case identification and recruitment

With our partners, we will identify and recruit up to 3 child welfare agencies (cases) that are in the early phases of START implementation (have not yet begun serving families) that are also willing to test out the CASPI. To explore how the CASPI is used under different conditions, we will purposefully maximize variation in our recruitment efforts (e.g. by recruiting an agency with an established partnership with a substance use provider as reported in the vignette experiment, an agency without an established partner, urban and rural agencies, etc.).

#### Pilot test and data collection

Leaders (executive director, program manager, designated START programmatic leader, etc.) from the participating child welfare agencies will be asked to take part in four meetings. First, during an *introductory meeting* with agency leadership, we will explain and address questions about the CASPI. Second, during an in-person *walk-through meeting* with designated agency leadership, the research team and agency leadership will collectively assess the context (deemed to be most salient in Aim 1), walk-through the decision analysis tool, and review the recommendations for collaborative strategies. We will use cognitive interviewing techniques [63, 64] to explore leaders’ reactions, and responses to the CASPI recommendations, the degree to which recommendations represent a substantial reorientation to typical agency collaboration, intentions to adopt them, and why. Finally, we will conduct 2 *follow up check-ins* with agency leaders at 2 and 4 weeks after the walk-through meeting. During these check-ins we will assess which recommendations were adopted, and why; how other CASPI contents were used; challenges that emerged; and recommendations. We also will explore the CASPI’s use as a stand-alone implementation tool (without the guidance issued through the walk-through meetings). All meetings will be audio recorded and transcribed; research team members will record their observations and reflections. We also will request that agencies share any MOUs or contracts.

#### Coding/analysis/reporting

Transcripts, and research team notes/reflections will be analyzed for each site iteratively by two independent coders using a modified grounded theory approach – modified in that we will orient our initial open coding process around “sensitizing concepts” including issues related to acceptability, appropriateness, and feasibility [65, 66]. The MOUs/contracts will be analyzed using the Aim 1 codebook. We will prepare case descriptions, and conduct within and across cases analyses [67].

### Analysis - data integration

We will integrate the quantitative and qualitative data during interpretation, which will be framed around several organizing questions (Table 3). Quantitative data will suggest the acceptability, appropriateness, and feasibility of the CASPI, while the in-depth descriptive case studies will highlight the CASPI’s practical utility.

**Table 3:** Aim 3 Organizing questions

quant (vignettes)	QUAL (case studies)
Acceptable: *Perceived appeal, or satisfaction*	
Do agency leaders like the CASPI? More acceptable than general support?	What elements are appealing (or not), why?
Appropriate: *Perceived relevance, compatibility, suitability*	
Do agency leaders think the CASPI is appropriate? More than general collaboration support?	What elements & recommendations are suitable (or not) for child welfare leaders’, why?
Feasible: *Perceived usefulness, likelihood of successful use*	
Do agency leaders think the CASPI is feasible/easy to use? More than general collaboration support?	What elements & recommendations are feasible (or not), why?

## Discussion

To improve implementation of START and other cross-system interventions that benefit those with complex service needs, there is a need to establish effective collaborative relationships across systems quickly. Our work has potential to enhance the effectiveness and efficiency of implementing cross-system interventions like START by helping leaders identify challenges, and effective collaborative strategies that address them. Our results and the CASPI will have potential applicability in other county or regional systems seeking to implement innovations that align services across systems.

This study offers the opportunity to advance implementation science in several ways. First, this study will advance our understanding of different collaboration forms as system-level implementation strategies. These strategies target the system structure by linking organizations strategically across systems, but also link the outer system with inner organizational contexts. Therefore, we anticipate that our results could also generate new insights about ‘bridging factors’ as described in the EPIS model [31, 68], and expand strategy taxonomies. Findings about the use and effectiveness of these strategies under different county contexts also have potential to fill substantial gaps in our knowledge about the role of the outer setting. This is especially important in social service agencies (like child welfare agencies) which are highly sensitive to expectations, resource availability, and needs in the external environment [30, 32, 69–71]. These findings will also contribute to collaboration scholarship which often disconnects studies of partnership formation and outcomes, and consequently has struggled to keep pace with collaboration practice [34].

Second, our study focuses heavily on rural service delivery systems. Much of our knowledge about implementation and services is based on research conducted in urban areas with robust resources and service delivery systems [72]. Rural systems experience unique service issues [73], with relatively few available behavioral health providers [74–77]. Waiting lists, local workforce shortages, travel distances, and fees further limit accessibility [78–80]. As a result, rural systems experience unique collaboration and implementation barriers. These systems offer an exceptional context for identifying the diversity of collaborative strategies to support implementation of cross-system interventions like START.

Ultimately, our study lays the groundwork for a Hybrid Type 2 implementation trial testing the effectiveness of CASPI for improving implementation, service, and client outcomes in a larger sample of counties. Because these collaborative strategies are applicable to other settings, future studies might test the effectiveness of collaborative strategies to implement cross-system interventions that facilitate service access and integration across other settings.

## Supplementary information

**Additional file 1:.** Ohio START Design, History, and Implementation

**Additional file 2:.** Ohio Sobriety, Treatment and Reducing Trauma (START) timeline narrative & case flow description

**Additional file 3:.** CONSORT checklist

## Data Availability

Not applicable.

## Abbreviations

CASPI: Collaborating Across Systems for Program Implementation
MOU: Memorandum of Understanding
MOUD: Medication for Opioid Use Disorders
NSSATS: National Survey of Substance Abuse Treatment Services
Ohio START: Sobriety Treatment Reducing Trauma
PCSAO: Public Children Services Association of Ohio
QCA: Qualitative Comparative Analysis
SAMHSA: Substance Abuse and Mental Health Services Administration
START: Sobriety Treatment and Recovery Teams

## Footnotes

1 Following a strong tradition of home-rule, child welfare services in Ohio are county administered. Service delivery systems are organized around 85 public county-based agencies that serve 88 counties.
