# Supplementary material for: Establishing cross-systems collaborations for implementation: protocol for a longitudinal mixed methods study
Source: Implement Sci. 2020 Jul 16;15:55. doi: 10.1186/s13012-020-01016-9 (PMC7364639; doi:10.1186/s13012-020-01016-9)
Supplement: Supplementary file 1 — Additional file 1:. Ohio START Design, History, and Implementation [file 13012_2020_1016_MOESM1_ESM.pdf]

Establishing Cross-Systems Collaborations for Implementation: Study Protocol for Implementation Decision Support

Supplemental File 1

Ohio START Design, History, and Implementation

The national START model (Sobriety Treatment and Recovery Teams) is a child welfare intervention for families affected by child maltreatment and substance use disorder. START is intended to help caregivers address substance use disorders and associated safety risks to their children so they can keep their children in their home (avoid having their children removed to foster care or other out-of-home placement) or be re-united with their children who were removed. There are seven components that guide practice (Table 1), and a clear timeline for identifying substance use disorders, referring parents to local treatment, engaging them in services through intensive peer recovery supports, and aligning service plans through shared decision making.

Table 1. Seven Components of the National START Model

1	Early identification of families affected by substance use disorders (screening)
2	Quick access to quality treatment
3	Increasing parent recovery services and engagement in treatment through peer support.
4	Focusing on family-centered services and parent-child relationships
5	Increasing oversight for parents and children
6	Sharing responsibility for parent accountability & program outcomes across service systems
7	Collaborating across service systems and with the courts.

Adopting and Adapting START for Ohio

In collaboration with the Ohio Attorney General's office, and several other partners, the Public Children Services Association of Ohio (PCSAO) adopted START in early 2017. The national model was adapted slightly to also address trauma exposure among children and parents and was renamed locally as Ohio Sobriety Treatment and Reducing Trauma (Ohio START). Ohio START retains the core components of the national START model and adds a trauma screening and referral component that is conducted concurrent with the substance use disorder screening and referral.

Drawing on consultation from the national START model developers at Children and Family Futures, and feedback from the Ohio START steering committee, PSCAO operationalized the Ohio START model. They used local child welfare terminology (Figure 1) to specify each component of Ohio START and expected timelines consistent with the national model with the goal of engaging the caregiver in at least four substance use disorder treatments sessions within 38 days of having a child maltreatment report screened-in. These steps include (Figure 2):

1. Conducting a substance use screening with parents (using the UNCOPE) within 14 days of having a child abuse/neglect (CA/N) report screened into the child welfare system (through the public child serving agency; PCSA) and referring caregivers who screen positive to the Ohio START program.

2. Conducting a shared decision-making meeting (SDMM) with the caregiver, child welfare caseworker, and family peer mentor within four days of the Ohio START program referral, where the caregiver is invited to participate. Those that agree are asked to sign a release of information (ROI) form that allows the child welfare system and substance use treatment provider to share case information.

3. Weekly face to face meetings with the family peer mentor and child welfare caseworker begins within 4 days of the SDMM and continue for the first 60-90 days of the case; meetings are held twice monthly afterwards.

4. Caregivers' substance use disorder (SUD) service needs are further assessed and referred to treatment. Treatment is intended to be evidence-based and tailored to needs.

5. Caregivers begin SUD treatment within four days of referral and receive 4 sessions in 12 days.

6. Children's trauma exposure is screened using the Child Trauma Assessment Center's Screening Checklist (CTAC) and children are referred for further assessment and treatment, as needed, within 30 days of the CA/N report being screened in (specific to Ohio START).

7. A family team meeting (FTM) is held with the family, family peer mentor, child welfare caseworker, behavioral health treatment provider, and other team members to align case plans within 30 days after the referral to START, and every three months after (or upon relapse or safety concern).

Figure 2. Ohio START Timeline

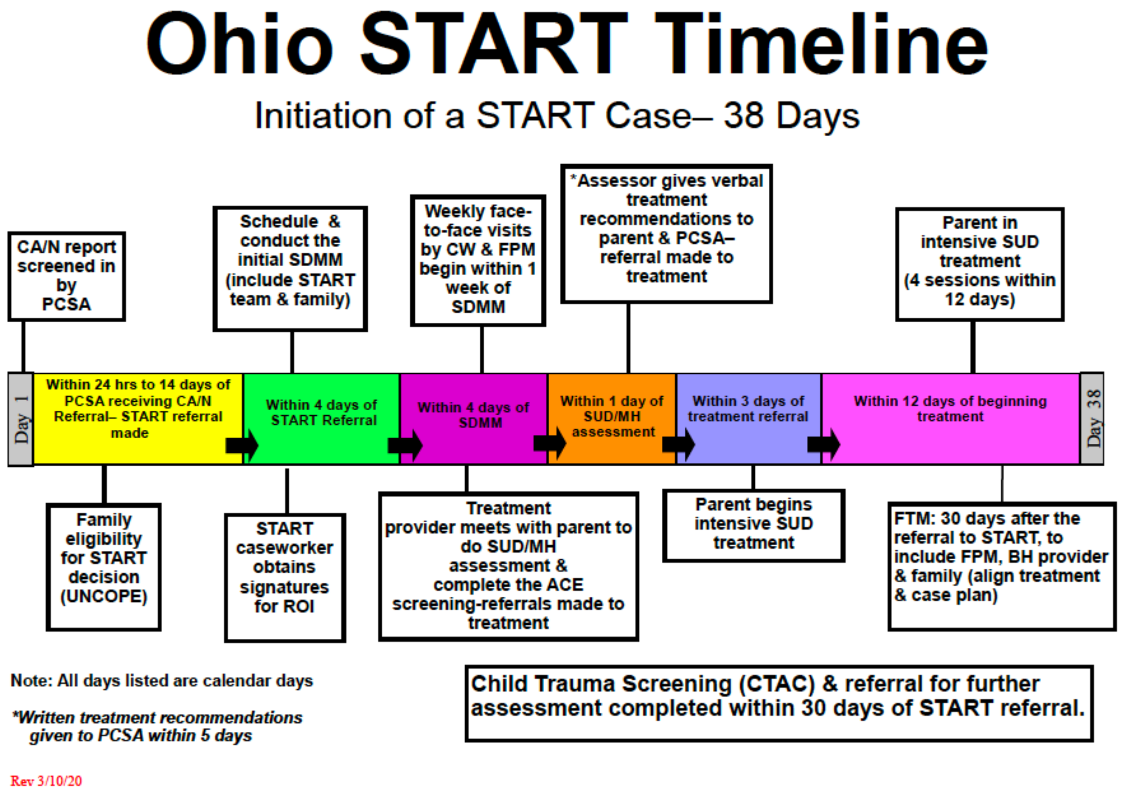

Ohio START Implementation

PCSAO began implementing Ohio START with an initial cohort of 17 counties in April 2017. Currently, Ohio START is led by and housed at the Public Children Services Association of Ohio (PCSAO). Implementation of Ohio START is funded by grants from the Ohio Department of Job and Family Services and the Ohio Department of Mental Health and Addiction Services

(OMHAS), the Victims of Crime Act (VOCA) grant program at the Ohio Attorney General's Office and a State Opioid Response (SOR) grant from OMHAS. In addition, Casey Family Programs, United HealthCare, Anthem, PhRMA and HealthPath Foundation of Ohio have provided grants to support the program. Information about the mission, history, project partners and resources is detailed on www.ohiostart.org.

Because this is a coordinated initiative funded by the State of Ohio and administered by PCSAO, all 17 counties follow a similar implementation plan drawing on the resources developed by and lessons learned during earlier pilots. Counties use a standard set of implementation strategies; using common language from implementation strategy taxonomies [1,2], these strategies are outlined in Table 2. In addition, all child welfare agencies are required to initiate a collaborative partnership with at least one local substance use treatment partner. However, the specific ways in which child welfare agencies are collaborating with substance use treatment organizations to implement Ohio START differs across counties.

Table 2. Standard Implementation Strategies for Initial Cohort of Ohio START Counties (n=17)

Strategy	Actor
Planning and Adapting to Context	
Develop an implementation plan	CWA, SU
Build buy-in among staff	CWA, SU
Identifying leadership (point of contact)	CWA
Training and Education	
Develop materials	PCSAO (contracted trainers)
Conduct ongoing trainings	
Financing	
Fund Ohio START implementation and services	Multiple
Quality Management	
Use OSU Needs Portal for ongoing quality monitoring	OSU
Provide technical assistance	PCSAO
*****Collaborative Strategy** (varies across counties)***	
Obtain formal written commitments delineating partners' role and efforts to align services & operations	CW/SU

CWA = Child Welfare Agency; OSU = Ohio State Evaluation Team; PCSAO = Public Children Services Association of Ohio; SU = Substance use treatment provider.

Since this initial cohort of 17 counties began implementing Ohio START, these efforts have been leveraged for an expansion of the program to 46 counties throughout the state of Ohio. Ohio START expanded to the second cohort (n=15 counties) in October 2018, a third cohort (n=14 counties) in October 2019. This study focuses on the first cohort, and the results will be used to strengthen collaboration in Cohorts 2 and 3.

References

1. Powell BJ, McMillen JC, Proctor EK, Carpenter CR, Griffey RT, Bunger AC, et al. A Compilation of Strategies for Implementing Clinical Innovations in Health and Mental Health. Med Care Res Rev. 2012; 69:123–57.

2. Powell BJ, Waltz TJ, Chinman MJ, Damschroder LJ, Smith JL, Matthieu MM, et al. A refined compilation of implementation strategies: results from the Expert Recommendations for Implementing Change (ERIC) project. Implement Sci. 2015;10:21.
