# Supplementary material for: Establishing cross-systems collaborations for implementation: protocol for a longitudinal mixed methods study
Source: Implement Sci. 2020 Jul 16;15:55. doi: 10.1186/s13012-020-01016-9 (PMC7364639; doi:10.1186/s13012-020-01016-9)
Supplement: Supplementary file 2 — Additional file 2:. Ohio Sobriety, Treatment and Reducing Trauma (START) timeline narrative & case flow description [file 13012_2020_1016_MOESM2_ESM.pdf]

Ohio Sobriety, Treatment and Reducing Trauma (START)

Timeline Narrative & Case Flow Description

This narrative is intended to provide supplemental detail to the START timeline diagram.

CA/N Report Screened in by Local Public Children Services Agency (PCSA)

- Day 1 of the START timeline is when the PCSA screens in the report for an assessment/investigation.

Family Eligibility for START Decision (UNCOPE)

- During the subsequent safety check, the county PCSAs will use the UNCOPE to identify caregiver substance abuse and eligibility for Ohio START.
- The children involved with this program must have at least one parent that has substance use disorder (SUD) and has mistreated the child. The child and the family will be identified by the PCSAs through the screening and assessment process.
- The goal is for identification and referral of families to Ohio START to be made within 14 days or less of the PCSA screening in the CA/N report. If there are circumstances that prohibited the referral within 14 days, a referral may be made up to 30 days from the date of the screened in report. No referrals may be accepted after 30 days.
- This includes families where the child remains in the home, where the child is removed from the home, and those referred to alternative response.
- **NOTE:** If parent(s) is in out-of-area SUD residential treatment, please refer to the additional Supporting Information Timeline & Case Flow Description for guidance.

START Caseworker Obtains Release of Information from Parents

- The family will be offered to participate in Ohio START and if they accept, they will sign the appropriate release of information.

Schedule & Conduct Initial Shared Decision-Making Meeting (SSDM)

- The initial shared decision-making meeting, also known as the "safety meeting" or "initial family team meeting", will occur within 4 days of the referral to and acceptance into the START program.
- The purpose of this meeting is collaborative development of the safety and service plans, keeping children in the home when safely possible.
- The PCSA caseworker will schedule an initial SDMM meeting with the family. The meeting should include the START PCSA caseworker, family peer mentor (FPM), and, if possible, treatment provider(s), and any other identified informal (i.e. family supports) and formal (i.e. community partners) supports.
- During this meeting, the START caseworker, FPM, and treatment provider(s) will meet the parent(s). The START supervisor may attend the meeting with the caseworker. The START program should be explained to the family, along with the different roles of the PCSA caseworker and FPM. Staff should engage the family in participating in a SUD/mental health (MH) assessment and the importance of following through with the recommendations regarding treatment. Any concerns/barriers (i.e. transportation, child care, legal assistance, etc.) to accessing the SUD/MH assessment and/or treatment should be discussed and a plan created to overcome them.

Weekly Face-to-Face Home Visits

- Families will receive intensive case management services with the case worker/family peer mentor dyad. The family will have weekly face-to-face visits from each for the first 60 days of the case.
- First home visit must be within one week of the initial shared decision-making meeting.
- For weekly face-to-face home visits, visits will occur by the caseworker and FPM; however, visitations of the dyad may differ by county (e.g. FPM and caseworker go on weekly visits together and FPM may go on additional visits alone or the caseworker and FPM conduct their required weekly visits separately but will go out together if needed). This may be decided at the county level and for each family. For purposes of fidelity to Ohio START:
  - Minimum of first visit must be caseworker and FPM together;
  - It is recommended that the caseworker and FPM continue to visit together, as needed, to establish roles and relationships;
  - Caseworker and FPM will both be expected to make weekly visits either together or separate;
  - Caseworkers and FPM will work together in terms of scheduling visits and
  - Caseworkers and FPM use a team approach with the family, whether visits are made together or separately.
- After 60 days, the caseworker will have a minimum of twice per month (biweekly) face-to-face contact with parent(s).
- After 90 days, the family peer mentor will have a minimum of twice per month (biweekly) face-to-face contact with parent(s).
  - **NOTE:** After 60 days, please refer to the Supporting Information Timeline & Case Flow Description document for additional guidance.

Treatment Provider Meets with Parent(s) to do SUD/MH Assessment & Complete ACE Screening

- Referral for the parent(s) for further assessment, should be made and assessment began within 4 days of the SDMM.
- The behavioral health provider will complete a comprehensive SUD/MH Assessment with the parent(s) and administer the adult trauma screen (ACE) as agreed to in the MOU between the agencies.
- Family peer mentor may accompany and assist the parent(s) with transportation to the initial behavioral health assessment.

Assessor Gives Verbal Treatment Recommendations to Parent & PCSA-Referral Made to Treatment

- The behavioral health assessor will give verbal treatment recommendations to the parent(s) and the PCSA within 1 day of assessment and written treatment recommendations provided to the PCSA within 5 days of assessment.
- Recommendations are based on American Society of Addiction Medicine (ASAM) Patient Placement Criteria and include treatment as needed for SUDs, MH, and trauma. The parent(s) will be referred for treatment within 1 day of the SUD/MH assessment.

Parent Begins Intensive SUD Treatment

- Within 3 days of the SUD/MH assessment, the parent(s) begin intensive treatment at an appropriate level of care. Intensive in START is defined as at least two sessions per week for the first two weeks, which is inherent in treatment level 2.0 or higher. For level 1.0 or less, treatment intensity may decline after the first two weeks if the need for continued or increased intensity is not noted.
- Quick access to SUD treatment is priority, but parents with co-occurring mental health/trauma issues should receive concurrent treatment when possible.

Parent in Intensive SUD Treatment

- The parent(s) should receive 4 treatment sessions within the first 12 days of treatment. The FPM should transport the parent(s) to the first 4 treatment sessions to help with treatment engagement. If the parent(s) does not want to be or cannot be transported by the FPM, the FPM should accompany the parent(s) to the treatment session or meet the parent(s) at the treatment facility to do a warm handoff and support the parent(s) as they begin treatment.

FTM: 30 days after the referral to START.

- FTMs should include, PCSAs, FPM, BH provider, and family, will be held at critical points within the case plan to align treatment and case plan such as:
    - Within 30 days after the referral to Ohio START
    - 3, 6, and 9 months into the case
    - Child safety concern/possible removal
    - At relapse
    - When crises occur
    - When treatment recommendations change
    - Prior to reunification
    - Within 30 days prior to case closure.

Note: Please refer to Supporting Information to Timeline Narrative & Case Flow Description regarding ongoing communication, support services, case closure and discharge from START.

Ohio Sobriety Treatment and Reducing Trauma (START) Supporting Information to Timeline Narrative & Case Flow Description

Participation in START if parent is in residential treatment, particularly if treatment is far away:

- Family will be eligible to participate in START if parent(s) enter residential treatment if the UNCOPE screen is conducted and the family agrees to participate in START prior to leaving for the residential treatment program.
- While the parent(s) are in the residential treatment program, the START team should engage with parent(s) as much as possible. This includes attending team meetings at the residential facility, meeting or having phone calls with parents(s) and coming up with a plan for parents(s) upon discharge, including establishing local supports upon return to the community for transition and a safety net.
- The required START activities (e.g. trauma screen, weekly visits, etc.) will begin upon parent(s) discharge from the residential treatment program.
- For parents(s) with short term jail sentences, the same protocol will apply.

After 60 Days of START Case Initiation:

- After 60 days for caseworkers and 90 days for family peer mentors, a minimum of twice per month (biweekly) face-to-face contact with parent(s), one of these biweekly contacts per month will occur in the home with the parent(s).
- Family peer mentor to visit children in foster care or relative placement with the social worker at least quarterly. Monthly is preferred when possible.
- The team will refer parents/families to parenting supports to address parenting in recovery, bonding between parents and children, and parenting skills.

Children's Trauma Assessment Center (CTAC) Trauma Screen:

- A trauma screen for the child(ren) will be administered within the first 30 days of referral to Ohio START.
- The PCSA will also ensure that children are screened for developmental and social-emotional delays, and are linked with needed services, with a special emphasis on referrals to trauma-informed services and supports for the child.
- The children services caseworker will conduct the child trauma screen CTAC and refer for further assessment to the behavioral health provider, as appropriate.

Ongoing Communications Will Include:

- Written weekly reports of treatment attendance/progress, as appropriate,
- Monthly direct line and teaming meetings, and
- Monthly case reviews.

Case Closure:

- In order to complete the Ohio START program, parent(s) must demonstrate sobriety, as proven through random drug screens. It is recommended that they have maintained sobriety for at least six months.
- *Discharge from START program prior to successful case closure if family is not participating in* START.
    - After 30 days of missed contacts with the START program, the agency should hold a family team meeting with all interested parties and discuss the attempts to engage the family and the plan for the case moving forward. The team can decide to leave the case in START to continue to try to engage family or the team can decide to transfer to another caseworker and end the family's participation in START. This will be individualized on a case by case basis and may depend on capacity to serve families in START.
